# Comprehensive assessment of phenolics and antiradical potential of *Rumex hastatus* D. Don. roots

**DOI:** 10.1186/1472-6882-14-47

**Published:** 2014-02-08

**Authors:** Sumaira Sahreen, Muhammad Rashid Khan, Rahmat Ali Khan

**Affiliations:** 1 Botanical Sciences Division, Pakistan Museum of Natural History, Garden Avenue, Shakarparian, Islamabad, Pakistan; 2 Department of Biochemistry, Faculty of Biological Sciences, Quaid-i-Azam University Islamabad, Islamabad 44000, Pakistan; 3 Department of Biotechnology, Faculty of Biological Sciences, University of Science and Technology, Bannu, Khyber Pakhtunkhwa 20100, Pakistan

**Keywords:** *Rumex hastatus*, Antioxidant activity, Solvent extraction, Phenolics, HPLC

## Abstract

**Background:**

Roots of *Rumex hastatus* (Polygonaceae) are traditionally used for the treatment of various ailments including liver and lung diseases. In this study, various solvent extracts of *R. hastatus* roots, like methanolic, n-hexane, ethyl acetate, chloroform, butanol and aqueous fractions were assessed through their antioxidant properties in vitro and determination of phenolic contents.

**Methods:**

Several parameters like DPPH˙, ABTS˙^+^, ˙OH, H_2_O_2_, superoxide free radical scavenging, iron chelating power, reducing power, β-carotene bleaching power, antioxidant capacity and total phenolics and flavonoids were evaluated. High Performance liquid Chromatography (HPLC) was also considered.

**Results:**

Though all the fractions exhibited dose dependant activity. The samples with the highest activity were the butanol and methanol fractions in all the assays except hydrogen peroxide radical scavenging assay where chloroform fraction showed the highest scavenging aptitude. On the other hand, aquous fraction showed significant beta carotene linoleic acid, while n-hexane and ethyl acetate fractions exhibited a lesser antioxidant activity in all the assays. HPLC revealed the presence of rutin, luteolin-7-glucoside, vitexin and luteolin.

**Conclusion:**

These results have to some extent substantiated the use of *R. hastatus* roots against different diseases, as an excellent basis of potential antioxidant due to the presence of sufficient amount of phenolics such as rutin and luteolin.

## Background

*Rumex hastatus* is suffrutescent richly branching shrub. It grows up to 90–120 cm tall and leaves with petioles of the same length as the blade; blade hastate, panicles terminal with erect-divergent, mostly simple branches, nut up to 2 mm long, brown, and long spindle-shaped roots. The plant is distributed in northern Pakistan, north east Afghanistan and south west of China, growing between 700-2500 m, sometimes grows as pure population [1]. Researches have reported that root and the whole plant of *R. hastatus* is used as medicine. It is laxative, alterative, tonic, used in rheumatism [2], skin diseases, bilious complaints, piles, bleeding of lungs etc. [3]. Plant is used as flavouring agent, carminative purgative and diuretic [4]. Literature demonstrates that *Rumex hastatus* is traditionally used in the treatment of sexually transmitted diseases including AIDS [5]. Oxidation is a source of energy for various bio processes but production of excessive oxygen free radicals causes oxidative damages that in turn initiate lipid peroxidation of protein and DNA [6,7]. All the livings are gifted with enzymatic and non enzymatic antioxidant contents which protect the body from oxidative free radicals and balance them [8]. In case of excessive free radicals large number of medicinal plants and fruits are used to minimize the effect due to the presence of bioactive antioxidant metabolites [9,10]. Recently large number of research groups has focus on medicinal plant and their bioactive ingredients to replace costly synthetic drugs [11]. Phenolic and polyphenolic constituents present in medicinal herbs leads retardation of lipid peroxidation and scavenging of free radicals [12,13]. Our previous reports reveals that medicinal plant and their bioactive compounds play important role in free radicals scavenging and is used as therapeutic agent in the treatment of various disorders [14-16]. In our previous studies, leaves of *R. hastatus* were evaluated for phenolic compounds [17], so it is hypothesisized that roots being an ethnopharmacological part of *R. hastatus,* might have potent antioxidant properties against free radicals but no reports are available on the antioxidant activity of *R. hastatus* roots. Therefore, present study was conducted to explore the total phenolic content, HPLC characterizations and antioxidant activities of methanolic extracts and its various fractions through various *in vitro* models.

## Methods

### Plant collection and preparation of extract

During September 2010 *R. hastatus* was collected from Quaid-i-Azam University Islamabad, and Abbottabad of Northern Pakistan, respectively after identification by world renowed taxonomist Professor Dr. Mir Ajab Khan. Voucher specimens with Accession No. 27813 (*R. hastatus*) were deposited at the Herbarium of Pakistan Museum of Natural History, Islamabad for future correspondence. After complete cleaning of *R. hastatus* roots were cut into pieces and dried under shade. The dried samples were processed as mentioned [17,18]. The dry extract obtained with each solvent was weighed and stored at 4°C for further investigations.

### Determination of total phenolics

The total phenolics were assayed by the spectrophotometric method [19] using Folin- Ciocalteu’s phenol reagent at 750 nm. Gallic acid standard solution (0–100 mg/l) used as standard as shown as milligrams of gallic acid equivalents (GAE) per gram of dried sample.

### Determination of total flavonoids

Total flavonoid content was determined following Yong *et al.*[20] at 506 nm. Rutin was used as a standard and expressed as milligrams of rutin equivalents per gram of dried sample.

### HPLC quantification of phenolic compounds

#### Sample preparation

50 mg of each fraction of both plants were extracted with 6 ml of 25% hydrochloric acid and 20 ml methanol for 1 hr. The obtained extract was filtered to a volumetric flask. The residue was heated twice with 20 ml of methanol for 20 min to obtain the extract. The combined extract was diluted with methanol to 100 ml. 5 ml portion of the solution was filtered and then was transferred to a volumetric flask and diluted with 10 ml of methanol. The sample (10 μL) was injected into the HPLC apparatus.

### Chromatographic conditions

The HPLC separation was performed using an Agilent HPLC system through column 20RBAX ECLIPSE, XDB-C18, (5 μm; 4.6 × 150 mm, Agilent USA) with UV–VIS Spectra-Focus detector, injector-auto sampler. The isocratic mobile phase, consisting of tetrahydro-furan/acetonitrile/ 0.05% phosphoric acid solution (20:3:77, v/v/v), was delivered at a flow-rate of1.0 mL/min with some amendments in time and wavelentgth [21]. Prior to use the mobile phase was filtered through 0.45 mm Millipore membrane filters and degassed by sonication in an ultrasonic bath. Detection wavelength was set at 280 nm and the column temperature was maintained at 25ºC with injection volume of 10 μL.

### Standard solution and calibration curves

Methanol stock solution containing rutin, luteolin, vitexin and luteolin-7-glucoside was prepared and diluted to appropriate concentrations for the construction of calibration curves. At least six concentrations of the solution were analyzed in triplicate, and then the calibration curves were constructed by plotting the peak area versus the concentration of each analyte detected by HPLC.

### The LOD and LOQ

The stock solution containing rutin, luteolin, vitexin and luteolin-7-glucoside was diluted to a series of appropriate concentrations with methanol, and an aliquot of the diluted solutions was injected into HPLC for analysis. The LOD and LOQ under the present chromatographic conditions were determined at S/N of 3 and 10, respectively.

### Precision, repeatability, and accuracy

Intra- and inter-day variations were chosen to determine the precision of the developed assay. The known concentrations of rutin, luteolin, and luteolin-7-glucoside were tested. For intra-day variability test, the solution was examined in duplicates for three consecutive days while for inter-day variability test, the mixed standards solution was analyzed for six times within one day, Variations were expressed as the RSD.

To confirm the repeatability, BRR and MRR samples were extracted, respectively, and analyzed by HPLC as mentioned above. The RSD was used as the measurement of repeatability.

Recovery test was used to evaluate the accuracy of the method. A known amount of standards was added to a certain amount of MRR and BRR, and then extracted and analyzed using the method described above. Three replicates were performed for the test. The recovery was calculated as follows:

$$\begin{array}{ll} \text{Recovery}\;\left(\%\right)= & 100\times \left(\text{amount}\;\text{found}-\text{original}\;\text{amount}\right)/\\ & \text{amount}\;\text{spiked}. \end{array}$$

### Identification and quantification

Identification of the different compounds was made by comparing their retention time and UV spectra with those of pure standards. Quantification was performed on the basis of external standard method. Peak Resolution value (R) was determined by using the following formula.

$$R={\mathrm{RT}}_{1}-{\mathrm{RT}}_{2})/\left[0.5\left(W_{1}+W_{2}\right)\right]$$

Where RT_1_ and RT_2_ and W_1_and W_2_ are the times and widths, respectively, of the two immediately adjacent peaks.

### Antioxidant assays

Each sample was dissolved in 95% methanol at a concentration1 mg/ml and then diluted to prepare the series concentrations for various antioxidant assays including DPPH [22], superoxide anion radical [23], total antioxidant efficacy [24], hydroxyl radical [25], hydrogen peroxide [26], ABTS radical cation [27] β-carotene bleaching [28], iron chelation [29] and reducing power scavenging activity was determined according to the method of Oyaizu [30].

### Statistical analysis

All assays were carried out in triplicates and results are expressed as mean ± SD. Statistical comparisons were done with the ANOVA test. Differences were considered to be significant at p < 0.05. The EC_50_ values were calculated using the Graph Pad Prism 5 software.

## Results

### Extraction yield, total phenolics and flavonoid contents of *R. hastatus* roots

The yield percent of methanol extract of roots of *R. hastatus* and its different fractions is shown in Table 1. The extraction yield of these sample varied from 4.5 ± 0.5% to 12.5 ± 1.45% with a descending order of BRR > MRR > ARR > CRR > ERR > HRR. The total phenolic compounds in BRR (128 ± 3.6 mg gallic acid equivalent/g fraction) while the minimum in HRR (8.9 ± 1.4 mg gallic acid equivalent/g dry fraction). Flavonoid contents varied appreciably and its highest concentration was obtained in BRR (42.6 ± 1.9 mg rutin equivalent/g dry fraction) however the lowest concentration was observed in HRR (4.2 ± 0.78 mg rutin equivalent/g dry fraction).

**Table 1:** **Total phenolics, flavonoid and extraction yield of methanol extract and soluble fractions of *R. hastatus *** **roots**

**Plant extracts**	**Total phenolics (mg GAE/g dry fraction)**	**Flavonoids (mg Rutin equivalent (mg RTE/g dry fraction)**	**Extraction yield (%)**
MRR	111 ± 3.7c	38.6 ± 2.2c	12.5 ± 1.45d
HRR	8.9 ± 1.4a	4.2 ± 0.78a	4.5 ± 0.5b
ERR	11.3 ± 1.6a	7.0 ± 0.52a	5.7 ± 0.66b
CRR	81.9 ± 2.1b	37.7 ± 2.7c	3.2 ± 0.22a
BRR	128 ± 3.6d	42.6 ± 1.9d	11.3 ± 1.11d
ARR	88.1 ± 2.5b	22.4 ± 1.8b	9.1 ± 0.99c

Each value in the table is represented as mean ± SD (n = 3).

Values in the same column followed by a different letter are significantly different (p < 0.05).

### HPLC quantification of flavonoids

**Figure 1:**
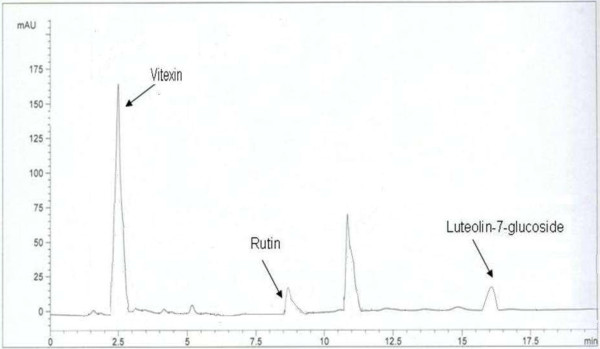
HPLC chromatogram of BRR fraction.

**Figure 2:**
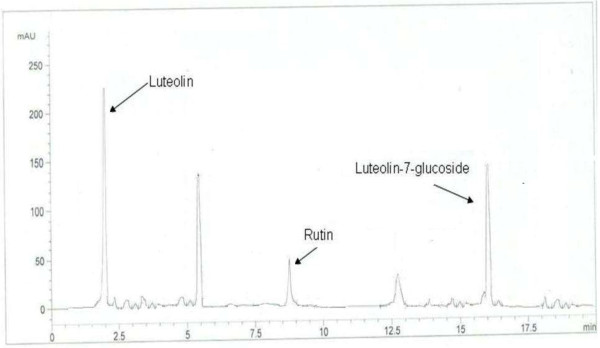
HPLC chromatogram of MRR fraction.

The HPLC-UV was preferred for the qualitative as well as quantitative analysis of different fractions of *R. hastatus* roots. Since the BRR and MRR exhibited the strongest antioxidant activity, they were quantified by assimilation of peak areas at 280 nm within runtime of 20 minutes (Figures 1 and 2). Experimental conditions were optimized to get the chromatograms with better resolution within a short resolution time and maximum UV absorption of sample.

### Linearity and detection limits

The response of the detector (relative peak area) was linearly dependent on the concentration of the sample i.e. 5.0-250 μg/mL for rutin, 10–100 μg/mL for luteolin-7-glucoside, 2.0-200 μg/mL for vitexin and 2.0-250 μg/mL for luteolin. Calibration curves for standard analytes at different concentrations were found to be linear and summarized in Table 2. Consequently, correlation coefficient values ranging from 0.9982 to 0.9998 revealed a good linearity response and sensitivity for developed method. The limits of detection (LOD’s) of rutin, luteolin-7-glucoside, vitexin and luteolin were 2, 5, 1 and 1 ng/mL while the LOQs obtained were 6, 12, 3 and 3 ng/mL, respectively. This indicated that the proposed method exhibits a good sensitivity for the quantification of four flavonoids in the RH roots.

**Table 2:** Retention time, linear range, calibration curve, Correlation coefficient, LOD and LOQ of four flavonoids determined by HPLC (UV)

**Compound**	**Retention time**	**Linear range (μg/ml)**	**Calibration equation**	**Corelation coefficient (r)**	**LOD (ng)**	**LOQ (ng)**
**Rutin**	8.7	5.0-250	y = 8.623 × 105x + 946.965	0.9998	2	6
**Luteolin-7-glucoside**	16.0	10-1000	y = 1.433x106x + 1064.141	0.9988	5	12
**Vitexin**	2.5	2.0-200	y = 3.357x105x + 1085.241	0.999	1	3
**Luteolin**	2.01	2.0-250	y = 1.429x106x + 3818.831	0.9982	1	3

### Precision and recovery

**Figure 3:**
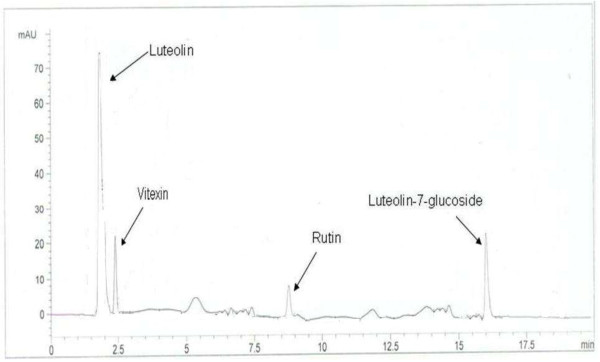
HPLC chromatogram of mixed standard solutions of four flavonoids.

To testify the precision and accuracy of the current method, RSD% values expressed as intra- and inter-day precisions and recoveries% of the standard analytes are determined in Table 3. The intra- and inter-day precisions were from 1.57% to 1.86% and 1.89% to 2.59% for the analytes, respectively. Hence, a good repeatability of the current method was noticed by measuring the relative peak areas of the standard analytes. To evaluate the accuracy of the proposed method. Recovery % values were obtained by the peak areas of the samples at three different concentrations of standard analytes. Table 3 also describes that the recoveries of the analytes were from 97% to 100.4% with RSD values of 1.53% to 2.23%. The content of the four analytes in the samples (MRR and BRR) was calculated by the corresponding regression equation. MRR sample contains the rutin, luteolin and luteolin-7-glucoside, while the BRR contains the rutin, vitexin and luteolin-7-glucoside. The HPLC chromatograms showed that the peak resolution values are greater than 1.0 that confirms the suitability and accuracy of the proposed method for the quantitative analysis of RH roots (Figure 3).

**Table 3:** Precision, Recovery and Content of four flavonoids in RH roots determined by HPLC (UV)

	**Precision**		**Recovery**		**Content (mg/g)**	
**Compound**	**Intra-day (RSD,%)**	**Inter-day (RSD,%)**	**Mean (%)**	**RSD (%)**	**MRR**	**BRR**
**Rutin**	1.57	1.95	98	1.53	1.2 ± 0.12	0.78 ± 0.02
**Luteolin-7-glucoside**	1.86	1.89	100.4	2.01	3.19 ± 0.24	0.87 ± 0.13
**Vitexin**	1.74	2.59	98.5	1.89	----	5.62 ± 0.47
**Luteolin**	1.69	2.52	97	2.23	6.12 ± 1.11	----

(----), not found.

Mean ± SD (n = 3).

The results suggest that the activity of BRR and MRR is attributed to phenolic compounds and in particular to rutin, luteolin, luteolin-7–O-glucoside and vitexin however, unknown (peaks) compounds may also involve in the antioxidant activities of RH roots.

### *In vitro* antioxidant assays

#### DPPH radical scavenging activity

**Figure 4:**
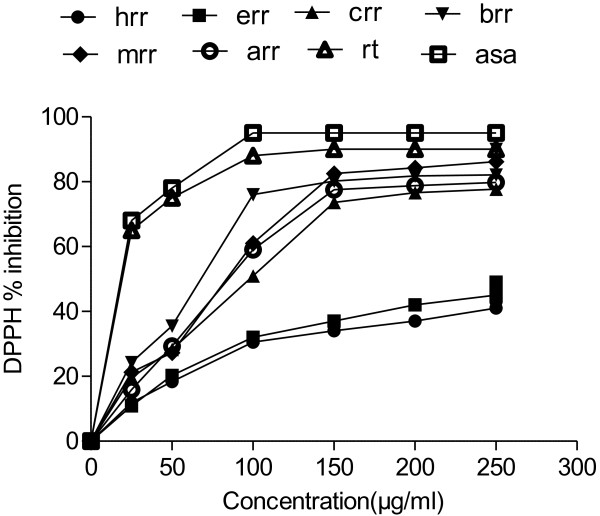
**DPPH radical scavenging activity of different extracts from the methanol extract of *R. hastatus *roots by different solvents at different concentrations.** Each value represents a mean ± SD (n = 3).

Figure 4 shows that the various fractions of *R. hastatus* roots extracts has DPPH free radical scavenging activity as BRR > MRR > AFC > CRR > ERR > HRR. The scavenging ability on DPPH radicals at 0.1 mg/ml of dried extract were 76.12 ± 2.11%, 61.13 ± 4.61%, 59.22 ± 2.34%, 50.23 ± 1.51%, 32.11 ± 2.13% and 30.51 ± 4.13% for BRR, MRR, ARR, CRR, ERR and HRR, respectively. The increase in activity at 0.25 mg/ml was 90.1 ± 2.13%, 86.2 ± 3.44%, 79.8 ± 1.19%, 77.7 ± 4.12%, 49.26 ± 1.34% and 43.21 ± 2.31% for the same order. The EC_50_ values of scavenging DPPH radicals for the BRR was 68.04 ± 1.7 μg/ml while for the HRR was > 246 μg/ml (Table 4).

**Table 4:** **Antioxidant effect (EC**_**50**_**) of methanol extract and its various fractions of *R. hastatus *** **roots**

**Plant extracts/chemical**	**EC50 μg/ml**							
	**Scavenging ability on DPPH radicals**	**Scavenging ability on super oxide radicals**	**Total antioxidant capacity**	**Scavenging ability on hydroxyl radicals**	**Scavenging ability on H**_**2**_**O**_**2 **_**radicals**	**Scavenging ability on ABTS radicals**	**β-carotene bleaching inhibition**	**Chelating power**
MRR	84.16 ± 3.2c	49.06 ±1.6c	21.21 ± 0.5a	61.87 ± 2.4c	90.21 ± 2.1c	128.01 ± 2.1c	126.67 ± 2.9d	333.11 ± 2.3b
HRR	>246e	>250f	>250d	>250e	> > 250d	> > 250e	>500f	> > 8000f
ERR	>241e	>250f	242.78 ± 5.4c	>248e	>250d	>250e	>500f	>8000f
CRR	99.23 ± 2.3d	62.28 ± 3.3e	45.85 ± 1.2b	85.29 ± 2.1d	75.11 ± 1.7b	136.09 ± 4.1c	138.09 ± 3.3e	1233.24 ± 4.5e
BRR	68.04 ± 1.7b	43.19 ± 2.4b	21.78 ± 0.8a	36.56 ± 1.6b	77.02 ± 1.5b	96.17 ± 1.12b	84.14 ± 2.6b	600.75 ± 4.1c
ARR	85.28 ± 1.9c	56.49 ± 0.9d	23.48 ± 0.4a	63.92 ± 1.9c	99.11 ± 3.3c	143.06 ± 1.8d	114.89 ± 3.6c	800.28 ± 2.2d
Ascorbic acid	19.59 ± 0.8a	22.36 ± 0.6a	20.79 ± 0.4a	29.78 ± 1.1a	23.04 ± 0.7a	65.23 ± 1.5a	-	-
Catechin		-	-	-	-	-	72.41 ± 2.5a	233.72 ± 1.9a
Rutin	19.31 ± 0.7a				27.10 ± 0.5a	-	-	-

Each value in the table is represented as mean ± SD (n = 3).

Values in the same column followed by a different letter are significantly different (p < 0.05).

-, not determined.

### Superoxide radical scavenging activity

The superoxide radical scavenging activity of different fractions of *R. hastatus* was compared with ascorbic acid ranging from 25–250 μg/ml. EC_50_ values in hydroxyl scavenging activities were in the order of BRR > MRR > AFC > CRR > ERR > HRR (Table 4). All of the fractions had a scavenging activity on the superoxide radicals in a dose dependent manner.

### Phosphomolybdate assay (Total antioxidant capacity)

Total antioxidant capacity depicts that different fractions of methanol extract of roots of *R. hastatus* can be ranked in the order of BRR > MRR > ARR > CRR > ELR > HRR. The EC_50_ values for the BRR and MRR was the same i.e. 21.78 ± 0.8 μg/ml, 21.21 ± 0.5 μg/ml, respectively while for the HRR it was >250 μg/ml (Table 4). The results obtained imply that the BRR and MRR have a remarkable ability to act as antioxidant.

### Hydroxyl radical scavenging activity

The hydroxyl radical scavenging activity can be ranked as BRR > MRR > ARR > CRR > ERR > HRR. Scavenging activity of all the extracts was found to be low when compared to ascorbic acid. The EC_50_ values of scavenging hydroxyl radicals for the BRR was 36.56 ± 1.6 μg/ml, while for the HRR was >250 μg/ml (Table 4).

### Hydrogen peroxide radical scavenging activity

Extracts from *R. hastatus* roots were capable of scavenging hydrogen peroxide in a concentration-dependent manner (25–250 μg/ml). The CRR and BRR fractions were equally potent in scavenging hydrogen peroxide, by 61.6 ± 1.21% and 57.1 ± 3.17% at a concentration of 100 μg/ml, respectively, while ERR and HRR were considerably less effective hydrogen peroxide scavengers (24.11 ± 1.22% and 27.03 ± 1.07%) at the same concentration. As compared with the EC_50_ values, the hydrogen peroxide- scavenging activities of CRR (75.11 ± 1.7 μg/ml) and BRR (77.02 ± 1.5 μg/ml) were comparable, and more effective (p < 0.05) than that of ERR (> > 250 μg/ml) and HRR (>250 μg/ml) (Table 4). The scavenging abilities on hydrogen peroxide were in descending order of CRR > BRR > MRR > ARR > ERR > HRR.

### ABTS radical scavenging activity

ABTS radical scavenging ability of samples can be ranked as BRR > MRR > CRR > ARR > ERR > HRR. The percentage inhibition was 87.01 ± 1.12% , 92.18 ± 0.08% and 82.03 ± 2.24% for the BRR, MRR and CRR, respectively while for ARR, ERR and HRR inhibition was 78.01 ± 3.19%, 47.43 ± 1.45% and 41.1 ± 2.6% at a concentration of 500 μg/ml. BRR exhibited the highest radical scavenging activities comparative to HRR and ERR. The EC_50_ values obtained for the BRR (96.17 ± 1.12 μg/ml) was significantly different (p < 0.05) from the EC_50_ values obtained for the ERR (>250 μg/ml) and HRR (> > 250 μg/ml), which were comparable (Table 4) with reference chemicals.

### β-carotene bleaching assay

With regard to the β-carotene bleaching assay, the antioxidant activity of samples can be ranked as BRR > ARR > MRR > CRR > ERR > HRR. At 0.1 mg/ml, β-carotene bleaching inhibitions were 54.11 ± 2.33% , 48.09 ± 1.44%, 40.78 ± 3.42%, 39.1 ± 2.56%, 18.15 ± 2.13% and 15.37 ± 2.51% for BRR, ARR, MRR, CRR, ERR and HRR, respectively. At 0.5 mg/ml the inhibition was increased to 81.34 ± 2.34%, 73.12 ± 1.19%, 78.62 ± 2.67%, 72.18 ± 1.36%, 34.49 ± 2.34% and 29.09 ± 1.45% for the above order. The EC_50_ values of BRR and ARR were 84.14 ± 2.6 μg/ml and 114.89 ± 3.6 μg/ml, respectively (Table 4) which was comparable with catechin.

### Iron chelating activity

**Figure 5:**
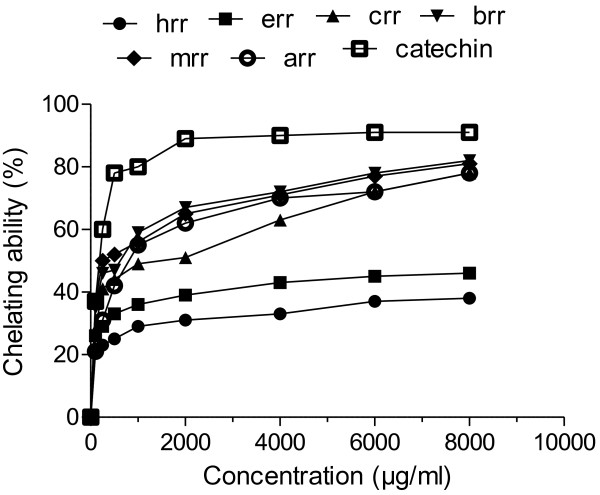
**Chelating power of different extracts from the methanol extract of *R. hastatus *roots by different solvents at different concentrations.** Each value represents a mean ± SD (n = 3).

Figure 5 shows that except ERR and HRR, other fractions showed significant chelation comparative to ascorbic acid. The sequence for chelating power was MRR > BRR > ARR > CRR > ERR > HRR. However, all the fractions presented much lower chelating power than catechin. The iron chelating data measured at different concentrations (100–8000 μg/ml) suggested that ferrous ion chelating effects of BRR and MRR would be somewhat beneficial to protect against oxidative damage. The EC_50_ values of iron chelating activity for the MRR was 333.11 ± 2.3 μg/ml while for the HRR it was >8000 μg/ml (Table 4).

### Reducing power activity

**Figure 6:**
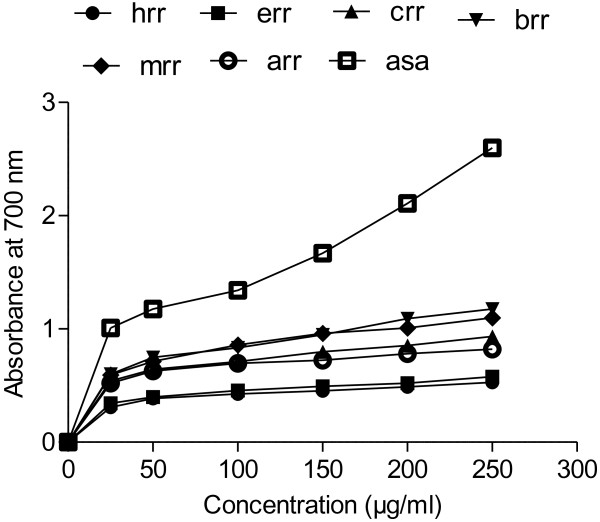
**Reducing power of different extracts from the methanol extract of *R. hastatus *roots by different solvents at different concentrations.** Each value represents a mean ± SD (n = 3).

Figure 6 shows the dose–response curves for the reducing powers of all extracts (25–250 μg/ml) from roots of *R. hastatus*. It was found that the reducing power increased with concentration of each sample. The sequence for reducing power was BRR > MRR > CRR > ARR > ERR > HRR. The various solvent fractions from roots of *R. hastatus* exhibited a good reducing power of 1.176 ± 0.14, 1.098 ± 0.17 and 0.931 ± 0.10 at 0.25 mg/ml for BRR, MRR and CRR, respectively. This data suggested that BRR and MRR have a remarkable ability to react with free radicals to convert them into more stable non-reactive species and to terminate radical chain reaction.

### Correlation of EC_50_ values of antioxidant activities and phytochemical contents

Table 5 showed the correlation between phyto-constituents and free radicals scavenging capabilities. EC_50_ values of radical scavenging activity various roots extracts of *R.hastatus* and the contents of bioactive compounds revealed a strong correlation.

**Table 5:** **Correlation**^**1 **^**between EC**_**50 **_**values of antioxidant activities and total phenolics and flavonoids of *R. hastatus *** **root extract and its various soluble fractions**

	**Correlation R**^**2**^	
**Assays**	**Phenolics**	**Flavonoids**
EC_50_ of DPPH radical scavenging ability	0.9476c	0.8479b
EC_50_ of superoxide radical scavenging ability	0.9277b	0.8508b
EC_50_ of antioxidant capacity	0.92172b	0.8238b
EC_50_ of hydroxyl radical scavenging ability	0.9605c	0.8478b
EC_50_ of hydrogen peroxide radical scavenging ability	0.8973b	0.8836b
EC_50_ of ABTS radical scavenging ability	0.9773c	0.9196b
EC_50_ of β-carotene bleaching inhibition	0.9296c	0.8389b
EC_50_ of chelating power	0.9238b	0.8391b

^1^*R.hastatus* roots methanol extract and its soluble fractions were used in the correlation.

Significantly different depicts that b (p < 0.01), c (p < 0.001).

## Discussion

As the plant derived polyphenols exhibit typical inhibitory trend against in vitro and in vivo oxidative reactions [31] due to redox properties therefore, it can be stated that tested plant samples may have important role to scavenge free radicals as they contain substantial quantity of phenolics and flavonoids. According to Sharififar *et al.*[32] dietary intake of flavonoid-containing foods was suggested to be of benefit as free radical stabilizers can replace the synthetic antioxidants by retarding lipid peroxidation. However, results obtained in the present study revealed that phenolics and flavonoids are main constituents of the plant having pharmacological tendency. Flavonoids are of considerable interest because of their possible inverse association with various chronic diseases like coronary heart disease [33] and several forms of cancer especially breast cancer [34]. In HPLC profile of *R. hastatus* roots, luteolin and rutin were recorded in higher amounts hence, could be consumed as a new source of bioactive compound. Qur studies revealed that type and amount of flavonoid compounds can be important evidence in the identification and evaluation of the best fraction. Data shows that HPLC profile of *R. hastatus* roots provide support to *in vitro* assays in which above mentioned fractions appeared to maintain strong antioxidant effects.

Antioxidant activity in an *in vitro* experiment is considered as the first step to point out potential health power of these fractions. Indeed, the study verified the role of phenolic and flavonoid contents extracted through different solvents against oxidative injuries. Thus, our results suggested that the extract can be exploited as an efficient and valuable antioxidant source, as some of the fractions showed highest scavenging ability than that of synthetic compounds. DPPH, a stable free radical, changes its color from violet to yellow after reduction by antioxidant or radical scavenger donating hydrogen- or electron [22]. The DPPH free radical has been commonly used to assess the antioxidative potential of plant extracts. It has been recommended that extracts rich in phenolics and flavonoids are involved in several biological activities including antioxidant ones. The study presented that power of fractions from roots of *R. hastatus* to scavenge DPPH radicals was associated with phytochemicals extracted by different solvents indicating that BRR and MRR can also possibly act as primary antioxidant. The superoxide anion is the more frequently produced free radical. Under oxidative stress, intense increase in superoxides results in cell and DNA damage which ultimately causes several pathological diseases [35]. It was therefore anticipated to calculate the relative ability of fractions to quench the superoxide free radicals. Several in vitro methods are accessible to generate superoxide free radicals [36]. In the present study, BRR and MRR behaved as strong superoxide anion quenchers among all the tested samples. The quenching ability of fractions may be the result of reactive concentration of bioactive compounds like phenolics and flavonoids and may help to put off oxidative damage of the major bio-molecules. Total antioxidant capacity of fractions/extracts has been estimated by phosphomolybdate method [24]. In this method, antioxidants reduce the Mo (VI) into Mo (V). The results obtained imply that BRR and MRR have notable antioxidant ability as compared to reference (ascorbic acid) antioxidant. This strong activity of various fractions of roots of *R. hastatus* might be a certificate for antioxidant behaviour of phenolic compounds. Reports of Sharififar *et al.*[32] also revealed that total antioxidant activity of medicinal plants is interlinked with flavonoids. Hence it offers an opportunity to develop less contemptible natural antioxidants. The hydroxyl radical is being concerned as highly reactive and detrimental species for about every molecule of biological system cause pathophysiological diseases. It has a potential to react with lipids, proteins and nucleotides of DNA causing oxidative damage. It was therefore proposed by Babu *et al*. [37] that hydroxyl radical scavenging ability is frankly allied with antioxidant activity. In the present study, BRR and MRR also reacted as strong hydroxyl quenchers. Thus, hydroxyl radical scavenging ability seems to be directly linked with prevention of lipid peroxidation and reduction of chain reactions.

Hydrogen peroxide itself is not very reactive, but it may produce hydroxyl free radicals that are very toxic to cells [38]. Inspite of this, previous researchers have focused on the hydrogen peroxide scavenging ability to find out the antioxidant status of the plant extract or pure compounds. Results showed the most valuable hydrogen peroxide scavenging ability of BRR and CRR as their values were analogous to that of reference compound. According to Hagerman *et al.*[39] phenolics with high molecular weight have additional capacity to quench free radicals like ABTS and their efficiency is more allied with number of aromatic rings and nature of hydroxyl group’s substitution as compare to functional groups. It can therefore be estimated that free radical (ABTS) scavenging action of BRR, MRR and CRR might be accredited with high molecular weight phenolics in addition to the flavonoids. β-carotene bleaching inhibition was accounted in different solvent extracts of dill (*Anethum graveolens*) flower [40]. The efficacy of *R. hastatus* root to hamper oxidation of linoleic acid emulsion is an indication of complex composition of fractions to interact with emulsion components. This data suggested that BRR and ARR have a notable propensity to scavenge free radicals that result in more stable non-reactive substances and to terminate radical chain reactions. Iron chelating data shows that various fraction possibly have talent to act against oxidative damage by chelating iron ions that may otherwise participate in decomposition of metal- catalyzed hydro peroxide and Fenton-Type reactions [41]. The iron sequestering frequency of various fractions measured at different concentrations proves that BRR and MRR may act as chelating agents against metallic ions. Accordingly it can be entailed that endogenous chelating agents like phenolics and flavonoids may be credited for iron chelating properties of various fractions. In addition, some phenolic compounds having well oriented functional groups possess the ability to protect against oxidative damage by chelating metal ions.

In the reducing power assay, donation of an electron is required to convert Fe^+3^/ferric cyanide complex to ferrous form that necessitate antioxidant. The total ferrous complex can be scrutinized by computing the development of Perl’s Prussian blue at 700 nm. Data suggested that BRR and MRR have a noteworthy power to react with free radicals by converting them into more stable substances and to stop radical chain reactions. Activity may be endorsed with the collective antioxidant effects of phytochemicals especially phenolics and flavonoids. The results indicate that phenolics and flavonoids are responsible for the antioxidant activities of fractions of *R. hastatus* roots, and decorated the importance of phenolic compounds in the antioxidant measures of plant fractions. A noticeable correlation among different tests confirmed the viability and consistency of selected antioxidant assay systems. Our results are in agreement with Ao *et al.*[42] who reported strong relationship with DPPH and ABTS as compare to β-carotene. Correlation studies of R. hastatus roots proved the role of phytochemicals especially phenolics and flavonoids in antioxidant potential of plant. The results suggest that the activity of BRR and MRR is attributed to phenolic compounds and in particular to rutin, luteolin, luteolin-7–O-glucoside and vitexin however, unknown (peaks) compounds may also involve in the antioxidant activities of RH roots.

In conclusion, the proposed HPLC method showed a good linearity, precision, repeatability, accuracy and recovery for the determination of four active compounds (rutin, luteolin, vitexin and luteolin-7-glucoside) and could be used for the quantitative analysis of RH roots. In this study, determination of phenolics and flavonoids, HPLC quantification and 9 antioxidant activity method with 6 extraction systems of different polarities, i.e., n-hexane, ethyl acetate, chloroform, butanol, methanol and water were compared. To our best knowledge this is the first record on the antioxidant potential of *R. hastatus* roots.

## Conclusion

We have demonstrated that *R. hastatus* roots extracts were capable of inhibiting and directly quenching free radicals to terminate the radical chain reaction which might be attributed to the presence of flavonoid contents such as luteolin, rutin, vitexin and luteolin-7-glucoside.

## Competing interests

The authors declare that they have no conflict of interests.

## Authors’ contributions

SS made a significant contribution to acquisition of data, analysis, drafting of the manuscript. MRK and RAK (ORCID ID: 0000-0003-0453-2090) have made a substantial contribution to conception and design, interpretation of data, drafting and revising the manuscript for intellectual content. All authors read and approved the final manuscript.

## Pre-publication history

The pre-publication history for this paper can be accessed here:

http://www.biomedcentral.com/1472-6882/14/47/prepub
